# Rectal swabs are a reliable proxy for faecal samples in infant gut microbiota research based on 16S-rRNA sequencing

**DOI:** 10.1038/s41598-019-52549-z

**Published:** 2019-11-05

**Authors:** Marta Reyman, Marlies A. van Houten, Kayleigh Arp, Elisabeth A. M. Sanders, Debby Bogaert

**Affiliations:** 1 Department of Paediatric Immunology and Infectious Diseases, Wilhelmina Children’s Hospital of University Medical Centre Utrecht, 3508 AB Utrecht, The Netherlands; 2 Spaarne Gasthuis Academy Hoofddorp and Haarlem, 2000 VB Haarlem, The Netherlands; 3 National Institute for Public Health and the Environment, 3720 BA Bilthoven, The Netherlands; 4 Medical Research Council/University of Edinburgh Centre for Inflammation Research, Queen’s Medical Research Institute, University of Edinburgh, EH16 4TJ Edinburgh, United Kingdom

**Keywords:** Microbiome, Microbiota, Paediatrics, Paediatric research

## Abstract

Rectal swabs are potentially a valuable method for monitoring the gut microbiome in research and clinical settings, where it is important to adhere to strict timing, or where acute sampling is needed. It is currently unknown whether rectal swabs give comparable results to faecal samples regarding microbiota community composition in neonates and infants. To study how well the two sampling methods correlate in infants, we compared the 16S-rRNA-based sequencing results of 131 paired rectal swabs and faecal samples collected from 116 infants at two timepoints in early life. The paired samples were highly comparable regarding both diversity and overall community composition, and strongly correlated on taxonomical level. We observed no significant nor relevant contribution of sampling method to the variation in overall gut microbiota community composition in a multivariable model. Our study provides evidence supporting the use of rectal swabs as a reliable proxy for faecal samples in infant gut microbiota research.

## Introduction

The interest in studying the gut microbiome in relation to health and disease is rapidly growing^1^. With continuing advances in rapid sequencing technology, monitoring of the gut microbiome in a clinical setting and conducting longitudinal microbiota studies into cause-effect relationships are becoming more feasible^2^. Currently, the routine sampling method for gut microbiota analysis is the collection of faeces^3^. However, it can be problematic to collect faeces within a narrow timeframe, as stool is not always readily available, especially in early life when the frequency of defaecation varies greatly^4^. Rectal swabs, on the other hand, can be collected easily and at any time, allowing flexible and consistent sampling between individuals and in relation to interventions. The collection of rectal swabs is already being applied for the screening of specific pathogens and multi-resistant organisms in the clinical setting^5^. Previous studies have shown that faecal samples and rectal swabs show satisfactory concordance when studying the gut microbiota in adults^6,7^. In the paediatric population, rectal swabs have been compared to faecal samples with respect to the detection of specific pathogens, such as norovirus^8^, but not yet on their performance in analysing the overall microbiota composition. The infant gut microbiota is very dynamic in the first weeks of life^9,10^, so it would be valuable to verify whether rectal swabs give a reliable representation of its composition in this period. If so, rectal swabs could be an ideal sampling method for the monitoring of treatment or study interventions on the neonatal ward or paediatric intensive care unit or in (longitudinal) population-based studies.

In our study, we compared the alpha and beta diversity between rectal swabs and faecal samples collected at the same time from the same individual at two sampling moments, and studied how well the sampling methods correlate on taxonomical level. The objective of our study was to determine whether rectal swabs are a good proxy for faecal samples in infant gut microbiota research.

## Results

After quality filtering, there were 131 closely paired faecal and rectal swab samples available from a subset of 116 neonates with suspected early onset neonatal sepsis participating in the ZEBRA study. A closely paired sample is defined as a matched faecal sample and rectal swab collected within a timeframe of 24 hours from an individual participant. Paired samples were collected at two timepoints: before start (timepoint 1) and after cessation (timepoint 2) of antibiotic treatment. For ethical reasons the start of antibiotic treatment could not be delayed whilst waiting for a faecal sample, so per our study protocol we collected rectal swabs from all neonates before the start of antibiotic treatment, and additionally, a faecal sample if the neonate had defecated before start of treatment as well, to avoid treatment bias. The number of times that both the faecal sample and rectal swab were collected before antibiotic administration had started was therefore restricted to 21/116 infants. At the second timepoint, we managed to obtain closely paired faecal samples and rectal swabs in 110/116 infants (median time difference between paired samples: 0 hours, range 0–24 hours). In a total of 23 cases the rectal swab was collected before the faecal sample (range 5 minutes to 24 hours), in 20 cases the rectal swab was collected after the faecal sample (range 15 minutes to 22,5 hours) and in 88 cases the rectal swab and faecal sample were collected at almost the same time, where in practice a trained physician collected the rectal swab first and a nurse collected the faeces shortly hereafter, the rectal swab being a stimulatory trigger for defaecation. The median age at sampling was 1 day for timepoint 1 and 6 days for timepoint 2. Further sample characteristics are detailed in Supplementary Table S1. Of the 116 infants included in this study, 15 had a paired sample available for both timepoint 1 and 2. Therefore, for analyses comparing sampling methods overall, we stratified per timepoint to take repeated measures into account. In order to answer our primary research question, namely if a rectal swab reliably reflects the fecal composition of an individual, we included all 131 paired samples, regardless of timepoint, for correlation analyses.

### Paired faecal samples and rectal swabs are comparable in alpha and beta diversity

The 262 samples analysed in this study represented 13,026,207 high quality Illumina Miseq sequences with a median Good’s coverage of 99.99% (range 99.72–100%). These sequences were annotated to 372 unique taxa. 270 taxa, representing 99.99% of all sequences, were present in both the faecal samples and rectal swabs. The combined relative abundance of the 52 taxa found only in faeces, and the 50 taxa found only in rectal swabs, was only 0.01%.

**Figure 1:**
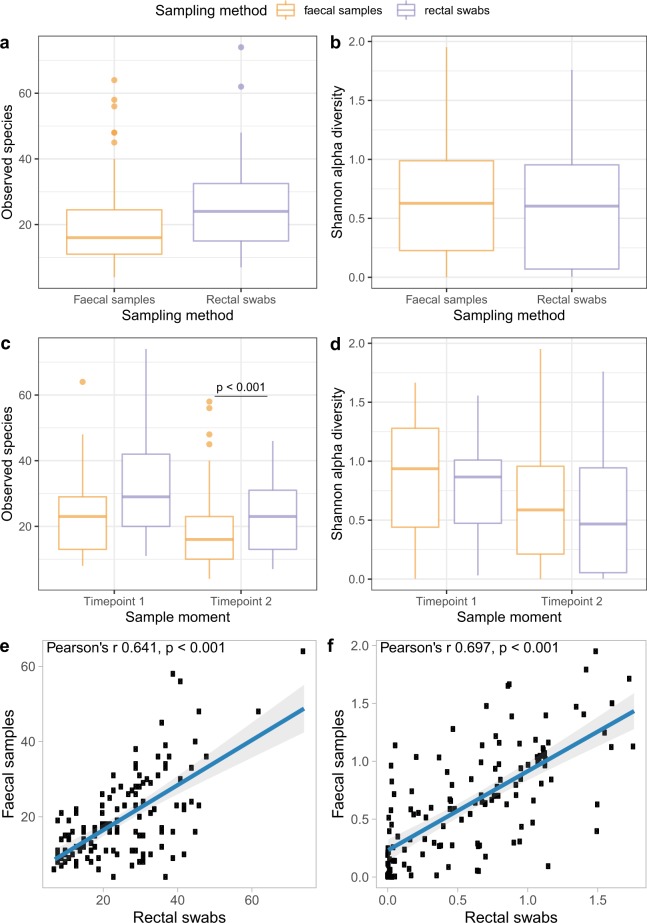
Alpha diversity measures. Differences are shown in observed species richness and Shannon diversity, respectively, between faecal samples and rectal swabs overall (**a**,**b**) and stratified per timepoint (**c**,**d**), to account for repeated measures of 15 participants from whom a paired sample was obtained at both timepoint 1 and 2. At timepoint 2 there was a significant difference in observed species richness between the faecal samples and rectal swabs (Wilcoxon test, median 16 versus 24, p < 0.001). No significant difference in Shannon diversity was found between the sampling methods. Boxplots with medians are shown; the lower and upper hinges correspond to the first and third quartiles (the 25^th^ and 75^th^ percentiles); the upper and lower whiskers extend from the hinge to the largest and smallest value no further than 1.5*IQR from the hinge; outliers are plotted individually. The observed species richness (**e**) and Shannon diversity (**f**) of the faecal samples are plotted against the observed species richness and Shannon diversity of their paired rectal swabs. A strong and significant correlation was found for both alpha diversity measures between the two sampling methods (Pearson’s r 0.641 and 0.697, respectively. Both p < 0.001).

Overall, we found no differences in alpha diversity between the faecal samples and rectal swabs as measured by using observed species richness and Shannon diversity (Fig. 1a,b). Since we found a correlation between alpha diversity and timepoint (Wilcoxon test p = 0.004), we also performed the analyses stratified per timepoint (Fig. 1c). At timepoint 1, we did not find a significant difference in observed species richness and Shannon diversity (Fig. 1d) between the sampling methods, though at timepoint 2 species richness was significantly higher in the rectal swabs compared to the faecal samples (Wilcoxon test, median 23 [range 7–42] versus 16 [range 4–58] Operational Taxonomical Units [OTUs], p < 0.001). When analysing the paired data, however, we found a strong correlation between paired faecal samples and rectal swabs for both species richness and Shannon diversity indices (Fig. 1e,f; Pearson’s r = 0.641, and 0.697, respectively: both p < 0.001).

**Figure 2:**
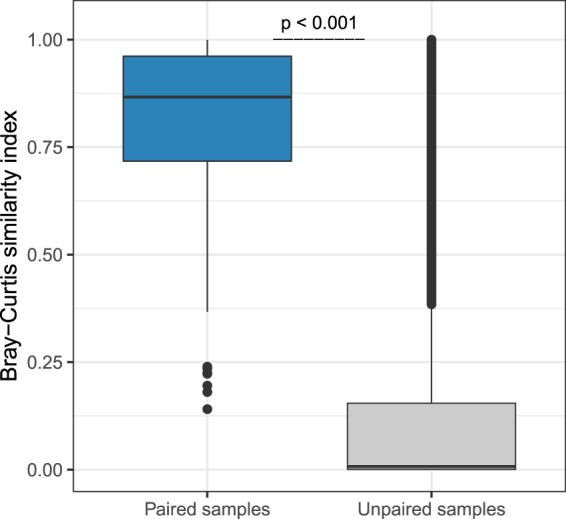
Concordance in microbiota community composition of paired rectal swabs and faecal samples. The concordance in microbiota community composition of paired faecal samples and rectal swabs was compared to the concordance of unpaired samples, using BC similarity (1-BC dissimilarity). A BC similarity index of 1 indicates an identical composition, while an index of 0 indicates the opposite. A significantly higher similarity was observed between paired samples (median BC similarity 0.866) than between unpaired samples (median BC similarity 0.007), as calculated by Wilcoxon. Boxplots with medians are shown; the lower and upper hinges correspond to the first and third quartiles (the 25^th^ and 75^th^ percentiles); the upper and lower whiskers extend from the hinge to the largest and smallest value no further than 1.5 * IQR from the hinge; outliers are plotted individually.

Regarding overall community composition, the two sampling methods did not differ at either timepoint, neither in an overall analysis, nor after stratification per timepoint (permutational multivariate analysis of variance [PERMANOVA]-test, R^2^ 0.006 with p = 0.994 at timepoint 1 and R^2^ 0.002 with p = 0.897 at timepoint 2; Supplementary Fig. 1). Furthermore, the composition of paired samples was significantly more similar than that of unpaired samples (Fig. 2; median Bray-Curtis [BC] similarity 0.866; versus median inter-individual similarity 0.007, p < 0.001). Neither the time between the collection of a paired faecal sample and rectal swab, nor the difference in reads between the sample types, was correlated with community composition similarity as measured by BC.

### Effect of sampling method compared to other clinical variables on microbial community composition

**Figure 3:**
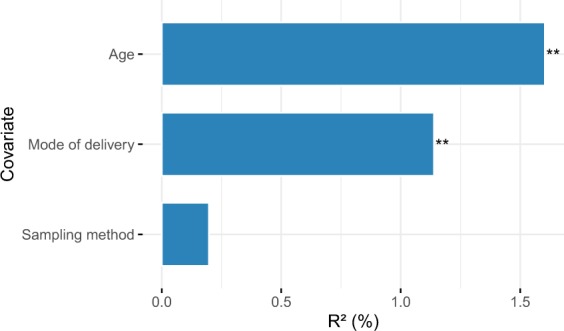
Variance explained in microbiota community composition by clinical variables. Covariates known to be associated with gut microbiota composition (age, delivery mode, feeding type) were first tested univariately with permutational multivariate analysis of variance (PERMANOVA)-tests using 1999 permutations, followed by a multivariable, temporal PERMANOVA, including sampling method to test what effect sampling method has on community composition in a clinical context. All samples were used with 1999 permutations and the strata parameter set to individual. The percentage of variance explained (R^2^ (%)) is plotted on the x-axis. Significant associations with microbiota composition (p = 0.002 for age and p = 0.005 for mode of delivery) are visualised in order of importance. Sampling method explained only a minor and non-significant component of the variance in community composition compared to all other parameters in the model (p = 0.835).

To evaluate the importance of sampling method for the overall observed variation in microbial community composition, we performed a multivariable PERMANOVA-test. First, we tested variables known to be associated with microbiota composition (age, delivery mode, feeding type) univariately and only included variables that showed a significant association in our study (age, delivery mode) in the multivariable model, along with sampling method. The contribution of sampling method to the variation in community composition was minimal and not significant (Fig. 3; R^2^ 0.002 p = 0.835), while participant’s age was the most important explanatory variable (R^2^ 0.016, p = 0.002).

### Paired faecal samples and rectal swabs correlate strongly on taxonomical level

**Figure 4:**
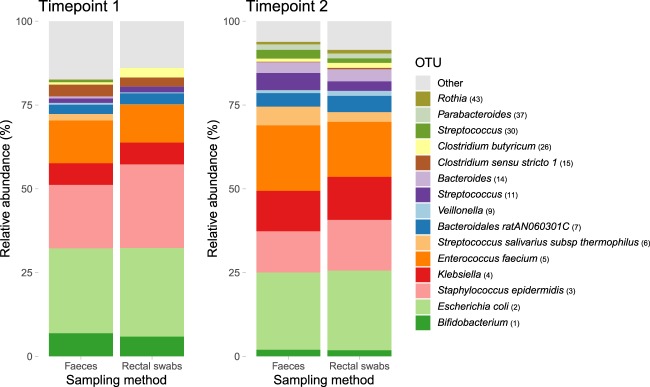
Relative abundance of the top 15 most abundant OTUs per timepoint and sampling method. Mean relative abundance of the top 15 most abundant Operational Taxonomical Units (OTUs) in this dataset, stratified per timepoint and sampling method.

The paired faecal samples and rectal swabs had a similar taxonomical composition with respect to the most abundant OTUs (Fig. 4). We performed Pearson correlation tests between the faecal samples and rectal swabs for all 198 testable OTUs, meaning that the 102 OTUs that were uniquely present in either the faecal samples or rectal swabs were excluded. The correlations of the top 15 most abundant taxa are shown in Table 1, all showing strong (Pearson’s r > 0.60) to very strong (Pearson’s r > 0.80) correlations. A total of 150 out of 198 testable OTUs correlated strongly and significantly in relative abundance between the paired samples with a median Pearson’s r of 0.92 (IQR 0.90–1.00, adjusted p-value < 0.05). Together, these 150 taxa had a combined relative abundance of 98.5% of all sequences observed in our dataset. The comprehensive results of the correlations of all OTUs can be found in Supplementary Table 2.

**Table 1:** Correlation of the top 15 most abundant OTUs between the two sampling methods.

OTU	mean RA faeces	mean RA rs	∆ mean RA	Pearson’s r
*Escherichia coli* (2)	23.39%	24.19%	−0.80%	0.95
*Enterococcus faecium* (5)	18.46%	15.61%	2.85%	0.96
*Staphylococcus epidermidis* (3)	13.38%	16.70%	−3.32%	0.76
*Klebsiella* (4)	11.17%	11.82%	−0.65%	0.99
Bacteroidales *ratAN060301C* (7)	3.84%	4.60%	−0.76%	0.94
*Streptococcus salivarius subsp thermophilus* (6)	5.00%	2.45%	2.55%	0.83
*Streptococcus* (11)	4.52%	2.66%	1.86%	0.68
*Bacteroides* (14)	2.79%	3.04%	−0.24%	0.98
*Bifidobacterium* (1)	2.79%	2.50%	0.29%	0.97
*Streptococcus* (30)	2.26%	1.15%	1.11%	0.70
*Parabacteroides* (37)	1.41%	1.22%	0.19%	0.99
*Clostridium butyricum* (26)	0.92%	1.71%	−0.79%	0.95
*Veillonella* (9)	0.81%	1.30%	−0.49%	0.68
*Rothia* (43)	0.65%	0.91%	−0.26%	0.98
*Clostridium sensu stricto 1* (15)	0.67%	0.77%	−0.10%	0.90

We studied, among others, the correlation of the relative abundance of the 15 most abundant Operational Taxonomical Units (OTUs) between the paired faecal samples and rectal swabs, using the Pearson’s correlation coefficient. For all 15 OTUs (with a combined relative abundance of 92.5%) we found a strong (Pearson’s r 0.60–0.79) to very strong (Pearson’s r 0.80–1) correlation between the paired faecal samples and rectal swabs. RA = relative abundance; rs = rectal swabs; ∆ mean RA = difference in mean RA between faecal samples and rectal swabs calculated for each OTU. A comprehensive list of results of the correlations of all OTUs can be found in Supplementary Table 2.

## Discussion

Rectal swabs are more flexible to collect than faecal samples for research and clinical purposes, and could therefore be a valuable tool in cases where it is important to adhere to strict sampling timeframes, as when sampling around time sensitive interventions. In this study, we showed that paired infants’ rectal swabs and faecal samples correlate well on alpha diversity, are comparable in overall community composition and correlate strongly on taxonomical level.

The observed species richness did not differ significantly between the two sampling methods at the earliest timepoint and correlated strongly between the paired faecal samples and rectal swabs. At the second timepoint, the observed species richness differed significantly between faecal samples and rectal swabs, even after filtering our OTU table of possible contaminants and also when repeating this analysis with rarefied data. However, this difference in diversity was contrary to what we expected: if at all different, we expected the rectal swabs, due to the smaller amount of material collected with this method, to pick up fewer species, but the opposite was the case, showing at least no taxa were missed with this type of sampling. Importantly, no differences in Shannon diversity were found between the sampling methods overall, or at either timepoint, and a clear correlation between paried samples was found. We also found an equal number of taxa that were unique to either sampling method (52 in faecal samples and 50 in rectal swabs), though the combined relative abundance of these was extremely low (<0.01%) and therefore less relevant for overall community structure. As a result, we found no difference in overall microbial community composition between the two sampling methods. Also, the concordance between the microbiota of a paired faecal sample and rectal swab collected from the same participant was high, confirming previous findings in adults^7^.

In a broader clinical context, we found it interesting to establish that sampling method did not significantly explain variation in microbiota composition, as opposed to known drivers such as age^11^, further supporting that rectal swabs are an appropriate proxy for faecal samples in infant gut microbiota studies. Unfortunately, we could not study the effect of antibiotics on composition in the multivariable model, because antibiotic treatment was colinear with timepoint in our study (sampling moments were before the start and after cessation of antibiotic treatment), and therefore age.

With respect to individual taxa, we found a high abundance of facultative anaerobic genera, such as *Escherichia coli*, and *Staphylococcus epidermidis* in the earliest samples, consistent with the description of normal early life gut microbiota development in previous studies,^12,13^. The paired faecal samples and rectal swabs showed a strong correlation for most bacteria abundantly present in the infant microbiota. The combined relative abundance of the taxa with a strong to very strong correlation (Pearson’s r > 0.60) between the sampling methods was above 98%, including predominant and clinically relevant taxa such as *Klebsiella* and *Enterococcus faecium*, which are known reservoirs for antibiotic resistance genes^14–16^, as well as taxa like *Bifidobacterium* which are associated with various beneficial functions^17^. Altogether, this underlines that rectal swabs are a very good proxy for faecal samples in microbiota analyses.

## Methods

### Study population and sample collection

To study whether rectal swabs are a good proxy for faecal samples in infant gut microbiota studies, we used a subset of 131 paired faecal and rectal swab samples from 116 children participating in the Dutch randomised controlled ZEBRA study. The ZEBRA study aims to evaluate the effects of antibiotic treatment indicated for (suspected) neonatal sepsis in the first week of life on the developing gut microbiota. Written informed consent was obtained from both parents. Ethical approval was granted by the national ethics committee in the Netherlands, METC Noord-Holland (committee on research involving human subjects, M014-024, NTR5119). The study was conducted in accordance with the European Statements for Good Clinical Practice.

Rectal swabs were collected using FaecalSwab™ kits (Copan Diagnostics, CA, USA) by trained physicians or research personnel before the start of antibiotic treatment (timepoint 1) and 24–48 hours after cessation of antibiotic therapy (timepoint 2). Faecal samples were obtained at the same timepoints, usually directly after the rectal swab, this being a stimulatory trigger for defaecation, and stored in sterile faecal containers by a nurse during hospital stay, or by the parents if the participant was already discharged at the later timepoint. All material was directly stored at −20 °C before being transferred (<2 weeks) to a −80 °C freezer until further laboratory processing. We only analysed paired samples that were obtained within 24 hours of one another, and in the case of timepoint 1 were also both obtained strictly before the start of antibiotic treatment.

### DNA isolation and sequencing

Bacterial DNA was isolated from faecal samples as previously described^18^. We used approximately 100 μl of faeces, 300 μl of lysis buffer, 500 μl zirconium beads and 500 μl of phenol, and performed an extra phenol/chloroform step. Samples collected on day 1 were presumed to have low bacterial abundance. Therefore, further adaptations were applied as described previously^19^, with the additional changes of using 150 μl instead of 100 μl of faeces (or 100 μl of material in the case of rectal swabs) and implementing an extra step with wash buffer 1. DNA blanks and a positive control consisting of a mix of up to three random faecal samples were used for quality control. The amount of bacterial DNA was determined by quantitative polymerase chain reaction (qPCR) as previously described^19^.

After amplifying the V4 hypervariable region of the 16S rRNA, quantification of the amount of amplified DNA per sample was executed with the dsDNA 910 Reagent Kit on the Fragment Analyzer (Advanced Analytical, IA, USA). Samples yielding insufficient DNA after amplification, defined as <0.5 ng/μl, were repeated with a higher concentration of template DNA. A mock control and three PCR blanks were included in each PCR plate. 16S rRNA sequencing was performed on the Illumina MiSeq platform (Illumina, Eindhoven, the Netherlands).

### Bioinformatic processing

The samples and their sequences described in this manuscript are part of a larger dataset existing of 2176 samples and controls, and together were processed using our in-house bioinformatics pipeline^20^. In short, we applied an adaptive, window-based trimming algorithm (Sickle, version 1.33) to filter out low quality reads, maintaining a Phred score threshold of 30 and a length threshold of 150 nucleotides^21^. Error correction was performed with BayesHammer (SPAdes genome assembler toolkit, version 3.5.0)^22^. Each set of paired-end sequence reads was assembled using PANDAseq (version 2.10) and demultiplexed (QIIME, version 1.9.1)^23,24^. Singleton and chimeric reads (UCHIME) were removed. OTU picking was conducted with VSEARCH abundance-based greedy clustering with a 97% identity threshold^25^. OTUs were annotated using the Naïve Bayesian RDP classifier (version 2.2) and the SILVA reference database^26,27^. This resulted in an OTU-table containing 18,951 taxa in total. We created an abundance-filtered dataset selecting OTUs present at a confident level of detection (0.1% relative abundance) in at least two samples^28^, hereafter referred to as our raw OTU-table. The raw OTU-table consisted of in total 730 taxa (0.49% sequences excluded with filtering). Next, we used both the prevalence and frequency methods of the *decontam* package^29^ to exclude possible contaminants, discarding 35 taxa, and thus retaining 695 taxa in total. The subset of paired samples studied here contained only 372 of these taxa.

### Statistical analyses

All analyses were performed in R version 3.4.3^30^ within RStudio version 1.1.383^31^ and figures were made using packages ggplot2^32^ and ggpubr^33^. The alpha diversity of the two sampling methods was compared using the observed species richness and Shannon diversity. When rarefying to a sequencing depth of 25,000 reads after filtering and decontamination (lowest quartile), or even 15,000 reads, the differences found using the raw data remained, so from here the raw (unrarefied) data was used for the comparisons and correlations in alpha diversity. Group differences were tested for using Wilcoxon tests. The correlation in alpha diversity between paired samples was calculated with Pearson.

The effect of sampling method on composition was analysed univariately with PERMANOVA-tests with 1999 permutations (adonis function; *vegan* package^34^) for all samples, and also stratified per timepoint, to prevent confounding by repeated measures. To visualise differences in composition we generated non-metric multidimensional scaling plots (nMDS; vegan package^34^). Ordinations were based on the Bray-Curtis (BC) dissimilarity matrix of relative abundance data with parameter trymax 10,000. To test whether paired faecal samples and rectal swabs (within child comparison) were more similar in microbiota composition than unpaired samples (between children comparison), we calculated the BC similarities (1 – BC dissimilarity) between all samples and compared the level of similarity between the paired and unpaired samples using Wilcoxon. For the paired samples, we also tested the correlation between composition and the difference in collection time and reads between the two sampling methods.

We performed a temporal, multivariable PERMANOVA-test (adonis2 function, *vegan* package^34^, 1999 permutations) to test whether sampling method contributes to the variation in overall gut microbiota community composition and how this relates to other known drivers of community composition. First, we tested covariates known to be associated with gut microbiota composition (age, delivery mode, feeding type) univariately with PERMANOVA-tests as described above. Only covariates that showed a significant association in the univariate analysis (age, delivery mode), were included in a multivariable model along with sampling method, whilst setting the strata parameter to individual.

Finally, we evaluated whether the sampling methods correlated on taxonomical level by calculating the Pearson correlation coefficient for all individual taxa based on their relative abundance. We also calculated the delta mean relative abundance between the two sampling methods to show the differences found.

P-values or, where applicable, adjusted p-values calculated using the Benjamini-Hochberg method^35^, <0.05 were deemed significant.

## Supplementary information

Supplementary Information

## Data Availability

Sequence data that support the findings of this study have been deposited in the NCBI Sequence Read Archive (SRA) database with BioProject ID PRJNA524461. The last 3 digits of the sample.id variable of the attributes are unique to a sample pair (with two exceptions: 387.006 is paired with 382.138 and 384.464 is paired with 383.572). The time variable refers to timepoint (1 = Timepoint 1, 2 = Timepoint 2). The mat variable refers to sampling method (2 = faeces, 5 = rectal swab).
