# Supplementary material for: Rectal swabs are a reliable proxy for faecal samples in infant gut microbiota research based on 16S-rRNA sequencing
Source: Sci Rep. 2019 Nov 5;9:16072. doi: 10.1038/s41598-019-52549-z (PMC6831562; doi:10.1038/s41598-019-52549-z)

Supplementary Information

Rectal swabs are a reliable proxy for faecal samples in infant gut microbiota research based on 16S-rRNA sequencing

Marta Reyman, Marlies A. van Houten, Kayleigh Arp, Elisabeth A.M. Sanders, Debby Bogaert

Supplementary Table S1: Sample characteristics. An overview of the sample numbers and characteristics is shown per sample moment. IQR = interquartile range.

	**Timepoint 1**	**Timepoint 2**
Total number of samples	**42**	**220**
Number of sample pairs*	21	110
Age of participants at sampling in days, median (IQR)		
faecal samples	1 (1-2)	6 (4-8.8)
rectal swabs	1 (1-2)	6 (4-9)
Time difference between paired sample collection in hours, median (IQR)	0 (0-0)	0 (0-2.5)
Reads per sample, median (IQR)		
faecal samples	34,142 (27,433- 40,674)	42,146 (29,216-55,482)
rectal swabs	41,252 (26,744- 55,923)	50,519 (42,058-63,011)

* Sample pair is defined as a matched faecal sample and rectal swab collected within 24 hours from an individual participant.

Supplementary Table S2: correlation of all OTUs between the two sampling methods. We studied the correlation of the relative abundance of all Operational Taxonomical Units (OTUs) between the paired faecal samples and rectal swabs, using the Pearson's correlation coefficient. RA = relative abundance; rs = rectal swabs; Δ mean RA = difference in mean RA between faecal samples and rectal swabs calculated for each OTU.

**OTU**	**mean RA faeces (%)**	**mean RA rs (%)**	**delta mean RA (%)**	**Pearson's r**	**adjusted p-value**
Escherichia coli (2)	23.39	24.19	-0.80	0.95	0.00
Enterococcus faecium (5)	18.46	15.61	2.85	0.96	0.00
Staphylococcus epidermidis (3)	13.38	16.70	-3.32	0.76	0.00
Klebsiella (4)	11.17	11.82	-0.65	0.99	0.00
Bacteroidales *ratAN060301C* (7)	3.84	4.60	-0.76	0.94	0.00
Streptococcus salivarius subsp thermophilus (6)	5.00	2.45	2.55	0.83	0.00
Streptococcus (11)	4.52	2.66	1.86	0.68	0.00
Bacteroides (14)	2.79	3.04	-0.24	0.98	0.00
Bifidobacterium (1)	2.79	2.50	0.29	0.97	0.00
Streptococcus (30)	2.26	1.15	1.11	0.70	0.00
Parabacteroides (37)	1.41	1.22	0.19	0.99	0.00
Clostridium butyricum (26)	0.92	1.71	-0.79	0.95	0.00
Veillonella (9)	0.81	1.30	-0.49	0.68	0.00
Rothia (43)	0.65	0.91	-0.26	0.98	0.00
Clostridium sensu stricto 1 (15)	0.67	0.77	-0.10	0.90	0.00
Bacteroides (31)	0.74	0.65	0.09	0.96	0.00
Lachnospiraceae (44)	0.65	0.68	-0.02	1.00	0.00
Streptococcus pyogenes (17)	0.46	0.76	-0.30	0.99	0.00
Peptostreptococcaceae (32)	0.41	0.80	-0.39	0.10	0.33
Streptococcus gallolyticus subsp macedonicus (16)	0.72	0.34	0.38	0.72	0.00
Corynebacterium (47)	0.45	0.57	-0.12	0.33	0.00
Haemophilus (38)	0.48	0.46	0.02	0.75	0.00
Prevotella (27)	0.22	0.59	-0.37	0.97	0.00
Bacteroides (33)	0.15	0.65	-0.50	0.78	0.00
Bifidobacterium animalis (36)	0.30	0.40	-0.11	1.00	0.00

Erysipelotrichaceae (34)	0.27	0.43	-0.16	1.00	0.00
Megamonas (46)	0.18	0.30	-0.12	0.99	0.00
Lactobacillus (40)	0.30	0.15	0.15	1.00	0.00
Clostridium sensu stricto 1 (23)	0.31	0.13	0.18	0.33	0.00
Streptococcus anginosus subsp whileyi (55)	0.32	0.07	0.25	0.69	0.00
Stenotrophomonas maltophilia (91)	0.23	0.12	0.11	1.00	0.00
Bacteroides (94)	0.21	0.12	0.09	1.00	0.00
Parasutterella (88)	0.06	0.26	-0.20	1.00	0.00
Veillonella (29)	0.16	0.15	0.01	1.00	0.00
Corynebacterium striatum (77)	0.02	0.28	-0.26	0.52	0.00
Ruminococcus gnavus CC55 001C (10)	0.10	0.12	-0.02	1.00	0.00
Gemella (120)	0.01	0.16	-0.14	0.99	0.00
Bifidobacterium (63)	0.13	0.03	0.10	0.87	0.00
Bacillales (147)	0.08	0.01	0.07	0.60	0.00
Parabacteroides goldsteinii dnLKV18 (133)	0.00	0.09	-0.08	1.00	0.00
Lactococcus lactis (78)	0.06	0.02	0.03	0.46	0.00
Phascolarctobacterium (219)	0.01	0.05	-0.04	1.00	0.00
Sutterella (134)	0.02	0.05	-0.03	0.72	0.00
Bacillus cereus (68)	0.01	0.06	-0.05	1.00	0.00
Lactobacillales (142)	0.00	0.06	-0.06	0.84	0.00
Tepidimonas (95)	0.02	0.05	-0.03	0.20	0.03
Bacteroides (58)	0.02	0.05	-0.03	0.99	0.00
Eubacterium sp CS1 Van (96)	0.05	0.00	0.05	0.81	0.00
Bacteroides (194)	0.03	0.01	0.02	0.98	0.00
Neisseria (121)	0.01	0.04	-0.03	0.10	0.31
Parabacteroides distasonis (207)	0.02	0.02	0.01	0.98	0.00
Aquabacterium (152)	0.01	0.03	-0.02	0.15	0.12
Bacteroides (249)	0.02	0.01	0.01	0.95	0.00
Acinetobacter (80)	0.03	0.01	0.02	1.00	0.00
Peptostreptococcaceae (50)	0.01	0.02	0.00	1.00	0.00
Nitriliruptor (126)	0.01	0.02	-0.01	0.24	0.01
Acinetobacter (143)	0.01	0.02	-0.01	0.35	0.00

Streptococcus (213)	0.03	0.00	0.02	0.14	0.15
Bifidobacterium animalis (311)	0.00	0.03	-0.03	1.00	0.00
Comamonadaceae (130)	0.01	0.02	-0.01	0.11	0.25
Prevotella bivia (102)	0.00	0.02	-0.02	0.99	0.00
Enterococcus (186)	0.02	0.00	0.02	0.46	0.00
Staphylococcaceae (246)	0.02	0.00	0.01	0.75	0.00
Bacteroides (236)	0.01	0.01	0.00	0.33	0.00
Burkholderia (156)	0.01	0.01	-0.01	0.29	0.00
Bilophila wadsworthia 3 1 6 (123)	0.02	0.00	0.01	0.02	0.93
Lactococcus (189)	0.02	0.00	0.01	0.19	0.04
Veillonella (76)	0.01	0.01	0.00	0.19	0.04
Acinetobacter (157)	0.01	0.01	0.00	0.05	0.73
Enhydrobacter (119)	0.01	0.01	0.00	0.06	0.59
Veillonella (87)	0.01	0.01	0.00	0.75	0.00
Lactobacillus reuteri (106)	0.02	0.00	0.02	0.10	0.34
Actinomyces (127)	0.01	0.01	0.00	0.95	0.00
Streptococcus (303)	0.02	0.00	0.01	0.43	0.00
Lactobacillus jensenii (271)	0.01	0.01	0.00	1.00	0.00
Actinomyces (292)	0.01	0.00	0.01	0.86	0.00
Bifidobacterium animalis (202)	0.01	0.01	0.00	1.00	0.00
Bacteroides (214)	0.01	0.01	0.00	1.00	0.00
Pseudomonas fluorescens (144)	0.00	0.02	-0.01	0.04	0.80
Bacteroides (423)	0.01	0.01	0.00	0.99	0.00
Bacillales (360)	0.01	0.00	0.01	0.62	0.00
Streptococcus (192)	0.01	0.00	0.01	0.31	0.00
Bacteroides (222)	0.01	0.01	0.00	0.92	0.00
Finegoldia (164)	0.00	0.01	-0.01	0.07	0.55
Collinsella (24)	0.00	0.01	-0.01	0.06	0.65
Escherichia Shigella (154)	0.01	0.01	0.00	0.63	0.00
Bacteroides (426)	0.01	0.00	0.01	0.99	0.00
Moraxella atlantae (514)	0.01	0.01	0.00	1.00	0.00
Streptococcus (338)	0.01	0.00	0.01	0.97	0.00
Lactobacillus salivarius (85)	0.00	0.01	0.00	1.00	0.00
Enterococcaceae (191)	0.01	0.00	0.01	0.92	0.00
Sphingobium (257)	0.01	0.00	0.01	-0.01	0.94

Bacteroides plebeius DSM 17135 (183)	0.00	0.01	-0.01	1.00	0.00
Bacteroides (70)	0.01	0.01	0.00	0.40	0.00
Actinomyces (73)	0.01	0.00	0.01	1.00	0.00
Erysipelotrichaceae (53)	0.00	0.01	0.00	0.92	0.00
Enterococcus (376)	0.01	0.00	0.00	0.40	0.00
Bacteroides (210)	0.00	0.01	0.00	0.90	0.00
Clostridium difficile 630 (345)	0.00	0.01	-0.01	0.74	0.00
Paracoccus (266)	0.00	0.01	0.00	-0.02	0.94
Corynebacterium aurimucosum ATCC 700975 (330)	0.01	0.00	0.00	1.00	0.00
Pasteurella pneumotropica (301)	0.01	0.00	0.01	0.07	0.51
Firmicutes (169)	0.01	0.00	0.01	1.00	0.00
Fusicatenibacter saccharivorans (20)	0.00	0.00	0.00	1.00	0.00
Caulobacteraceae (520)	0.01	0.00	0.01	0.99	0.00
Lachnospiraceae (83)	0.01	0.00	0.00	1.00	0.00
Klebsiella (200)	0.01	0.00	0.00	0.67	0.00
Enterobacteriaceae (205)	0.01	0.00	0.01	1.00	0.00
Bifidobacterium (12)	0.01	0.00	0.01	0.23	0.01
Veillonella sp DNF00869 (201)	0.01	0.00	0.00	0.14	0.15
Leuconostoc (275)	0.01	0.00	0.01	-0.01	0.94
Aggregatibacter (356)	0.00	0.01	0.00	0.20	0.03
Staphylococcaceae (452)	0.01	0.00	0.00	0.74	0.00
Peptostreptococcaceae (396)	0.00	0.01	0.00	0.81	0.00
Bacteroides (324)	0.00	0.00	0.00	0.94	0.00
Peptostreptococcus (98)	0.00	0.01	-0.01	0.14	0.13
Bifidobacterium (161)	0.00	0.01	0.00	1.00	0.00
Corynebacterium freneyi (429)	0.00	0.00	0.00	0.33	0.00
Clostridium difficile 630 (359)	0.00	0.00	0.00	0.57	0.00
Bacteroides (48)	0.00	0.01	-0.01	0.40	0.00
Coriobacteriaceae bacterium WAL 18889 (151)	0.00	0.00	0.00	0.01	0.94
Staphylococcus (507)	0.00	0.00	0.00	0.43	0.00
Streptococcus gallolyticus subsp macedonicus (464)	0.00	0.00	0.00	0.98	0.00

Staphylococcus (427)	0.00	0.00	0.00	0.21	0.02
Chryseobacterium (448)	0.00	0.00	0.00	-0.01	0.94
Lactobacillus (371)	0.00	0.00	0.00	0.79	0.00
Staphylococcus (489)	0.00	0.00	0.00	0.17	0.06
Staphylococcaceae (529)	0.00	0.00	0.00	0.77	0.00
Streptococcus (537)	0.00	0.00	0.00	0.06	0.63
Bacteroides (645)	0.00	0.00	0.00	0.98	0.00
Actinomyces sp 2002 2301122 (203)	0.00	0.00	0.00	0.50	0.00
Bifidobacterium (272)	0.00	0.00	0.00	1.00	0.00
Bifidobacterium bifidum NCIMB 41171 (13)	0.00	0.00	0.00	0.94	0.00
Bifidobacterium (336)	0.00	0.00	0.00	0.92	0.00
Bacteroides (248)	0.00	0.00	0.00	1.00	0.00
Gardnerella vaginalis 0288E (71)	0.00	0.00	0.00	0.31	0.00
Serratia marcescens (575)	0.00	0.00	0.00	0.14	0.15
Bifidobacterium (146)	0.00	0.00	0.00	0.69	0.00
Veillonella (115)	0.00	0.00	0.00	0.23	0.01
Bacteroides (400)	0.00	0.00	0.00	0.99	0.00
Bacilli (471)	0.00	0.00	0.00	0.71	0.00
Enterobacteriaceae (312)	0.00	0.00	0.00	0.74	0.00
Bacteroides (603)	0.00	0.00	0.00	0.99	0.00
Enterobacteriaceae (632)	0.00	0.00	0.00	0.97	0.00
Corynebacterium (641)	0.00	0.00	0.00	-0.01	0.94
Micrococcus luteus (545)	0.00	0.00	0.00	0.01	0.94
Streptococcus urinalis (389)	0.00	0.00	0.00	1.00	0.00
Flavonifractor (111)	0.00	0.00	0.00	1.00	0.00
Anaerococcus tetradius (329)	0.00	0.00	0.00	0.46	0.00
Janthinobacterium lividum (430)	0.00	0.00	0.00	0.02	0.94
Sutterella (605)	0.00	0.00	0.00	1.00	0.00
Bacillales (615)	0.00	0.00	0.00	0.46	0.00
Corynebacterium (328)	0.00	0.00	0.00	0.58	0.00
Corynebacterium (321)	0.00	0.00	0.00	0.64	0.00
Veillonella (300)	0.00	0.00	0.00	0.94	0.00
Corynebacteriaceae (504)	0.00	0.00	0.00	0.03	0.85
Staphylococcus (588)	0.00	0.00	0.00	0.47	0.00

Enterobacteriaceae (626)	0.00	0.00	0.00	0.98	0.00
Phascolarctobacterium (165)	0.00	0.00	0.00	0.32	0.00
Moryella (60)	0.00	0.00	0.00	1.00	0.00
Leuconostoc mesenteroides (282)	0.00	0.00	0.00	0.92	0.00
Dermacoccus (382)	0.00	0.00	0.00	-0.01	0.94
Acinetobacter ursingii ANC 3649 (388)	0.00	0.00	0.00	-0.01	0.94
Halomonas (494)	0.00	0.00	0.00	0.02	0.94
Bifidobacterium animalis (647)	0.00	0.00	0.00	1.00	0.00
Pseudoxanthomonas (667)	0.00	0.00	0.00	-0.02	0.94
Peptoniphilus (129)	0.00	0.00	0.00	-0.03	0.93
Methylobacterium radiotolerans (437)	0.00	0.00	0.00	-0.01	0.94
Bacteroides (535)	0.00	0.00	0.00	1.00	0.00
Dermabacter (335)	0.00	0.00	0.00	1.00	0.00
Streptococcus (425)	0.00	0.00	0.00	-0.01	0.94
Corynebacterium sp 1145 (518)	0.00	0.00	0.00	0.98	0.00
Raoultella ornithinolytica (495)	0.00	0.00	0.00	1.00	0.00
Corynebacterium propinquum (408)	0.00	0.00	0.00	-0.01	0.94
Bacteroides (681)	0.00	0.00	0.00	0.99	0.00
Bifidobacterium (233)	0.00	0.00	0.00	1.00	0.00
Rhizobium (561)	0.00	0.00	0.00	-0.02	0.94
Lactobacillus (42)	0.00	0.00	0.00	1.00	0.00
Vogesella (587)	0.00	0.00	0.00	-0.02	0.94
Streptococcus (521)	0.00	0.00	0.00	0.25	0.01
Blautia (8)	0.00	0.00	0.00	0.28	0.00
Plesiomonas (485)	0.00	0.00	0.00	0.94	0.00
Enterobacteriaceae (674)	0.00	0.00	0.00	1.00	0.00
Lactobacillus paracasei (18)	0.00	0.00	0.00	0.22	0.02
Bifidobacterium animalis (591)	0.00	0.00	0.00	1.00	0.00
Roseomonas (684)	0.00	0.00	0.00	-0.01	0.94
Bacteroides (380)	0.00	0.00	0.00	0.49	0.00
Bacteroides (482)	0.00	0.00	0.00	1.00	0.00
Ruminococcaceae (243)	0.00	0.00	0.00	0.05	0.67

Methylophilus (451)	0.00	0.00	0.00	0.11	0.26
Bifidobacterium animalis (293)	0.00	0.00	0.00	1.00	0.00
Bifidobacterium (223)	0.00	0.00	0.00	0.95	0.00
ratAN060301C (268)	0.00	0.00	0.00	0.54	0.00
Bifidobacterium animalis (560)	0.00	0.00	0.00	1.00	0.00
Alistipes (215)	0.00	0.00	0.00	0.99	0.00
Turicibacter (220)	0.00	0.00	0.00	-0.01	0.94
Staphylococcus (565)	0.00	0.00	0.00	0.24	0.01
Acinetobacter calcoaceticus (612)	0.00	0.00	0.00	-0.01	0.94
Bifidobacterium breve (473)	0.00	0.00	0.00	0.46	0.00
Aerococcus christensenii (61)	0.00	0.00	0.00	-0.01	0.94
Bacteroides (635)	0.00	0.00	0.00	0.97	0.00
Bacteroides (663)	0.00	0.00	0.00	1.00	0.00
Bacteroides (658)	0.00	0.00	0.00	1.00	0.00
Veillonella (502)	0.00	0.00	0.00	0.97	0.00
Peptostreptococcaceae (309)	0.00	0.00	0.00	-0.01	0.94
Ruminococcus gnavus CC55 001C (366)	0.00	0.00	0.00	1.00	0.00
Enterobacteriaceae (199)	0.00	0.00	0.00	0.13	0.19
Blautia (25)	0.00	0.00	0.00	0.02	0.94
Anaerostipes (22)	0.00	0.00	0.00	0.84	0.00
Bacteroides (242)	0.00	0.00	0.00	1.00	0.00
Peptoniphilus (413)	0.00	0.00	0.00	1.00	0.00
Varibaculum (117)	0.00	0.00	0.00	0.59	0.00
Subdoligranulum (136)	0.00	0.00	0.00	1.00	0.00
Anaerococcus (105)	0.00	0.00	0.00	-0.02	0.94
Bifidobacterium animalis (182)	0.00	0.00	0.00	1.00	NA
Lachnospiraceae (670)	0.00	0.00	0.00	1.00	NA
Sutterella (197)	0.00	0.00	0.00	0.28	0.00
Paracoccus (297)	0.00	0.00	0.00	0.46	0.00
Atopobium (174)	0.00	0.00	0.00	-0.01	0.94
Clostridium sensu stricto 1 (124)	0.00	0.00	0.00	-0.01	0.94
Subdoligranulum (45)	0.00	0.00	0.00	0.04	0.77
Bifidobacterium breve (634)	0.00	0.00	0.00	1.00	0.00
Subdoligranulum (81)	0.00	0.00	0.00	0.46	0.00

Anaerococcus (476)	0.00	0.00	0.00	-0.01	0.94
Anaerococcus (418)	0.00	0.00	0.00	-0.01	0.94
Lactobacillus delbrueckii subsp bulgaricus (190)	0.00	0.00	0.00	-0.01	0.94
Clostridium sensu stricto 1 (557)	0.00	0.00	0.00	-0.01	0.94
Clostridium sensu stricto 1 (462)	0.00	0.00	0.00	0.75	0.00
Phascolarctobacterium (317)	0.00	0.00	0.00	0.94	0.00
Eggerthella (178)	0.00	0.00	0.00	0.98	0.00
Bacteroides (247)	0.00	0.00	0.00	0.90	0.00
Xanthomonadales (274)	0.00	0.00	0.00	0.94	0.00
Lachnospiraceae (86)	0.00	0.00	0.00	0.37	0.00
Clostridium sensu stricto 1 (548)	0.00	0.00	0.00	0.74	0.00
Bacteroides (470)	0.00	0.00	0.00	0.77	0.00
Corynebacterium (460)	0.00	0.00	0.00	0.56	0.00
Faecalibacterium (21)	0.00	0.00	0.00	0.63	0.00
Streptococcus gallolyticus subsp macedonicus (350)	0.00	0.00	0.00	0.63	0.00
Clostridium sensu stricto 1 (621)	0.00	0.00	0.00	0.93	0.00
Prevotella melaninogenica (639)	0.00	0.00	0.00	-0.01	0.94
Negativicoccus sp S5 A15 (260)	0.00	0.00	0.00	-0.01	0.94
Dermabacter (617)	0.00	0.00	0.00	-0.01	0.94
Veillonella (454)	0.00	0.00	0.00	0.73	0.00
Bacteroides fragilis CL03T00C08 (291)	0.00	0.00	0.00	0.18	0.05
Alloscardovia (643)	0.00	0.00	0.00	1.00	0.00
Enterobacteriaceae (264)	0.00	0.00	0.00	0.88	0.00
Thermus (167)	0.00	0.00	0.00	-0.01	0.94
Bifidobacterium (347)	0.00	0.00	0.00	-0.01	0.94
Pantoea agglomerans (230)	0.00	0.00	0.00	-0.02	0.94
Veillonella (465)	0.00	0.00	0.00	0.19	0.04
Ruminococcus bromii L2 63 (62)	0.00	0.00	0.00	0.14	0.14
Candidatus Nitrososphaera (542)	0.00	0.00	0.00	1.00	0.00
Ferriphaselus (481)	0.00	0.00	0.00	-0.01	0.94
Bifidobacterium (228)	0.00	0.00	0.00	1.00	0.00
Peptostreptococcaceae (75)	0.00	0.00	0.00	-0.02	0.94

Eubacterium hallii DSM 3353 (52)	0.00	0.00	0.00	1.00	0.00
Bifidobacteriaceae (344)	0.00	0.00	0.00	1.00	0.00
Veillonella (555)	0.00	0.00	0.00	-0.01	0.94
Butyricimonas (503)	0.00	0.00	0.00	1.00	0.00
Blautia (79)	0.00	0.00	0.00	-0.01	0.94
Bacteroides (493)	0.00	0.00	0.00	1.00	0.00
Escherichia Shigella (469)	0.00	0.00	0.00	0.31	0.00
Gardnerella (332)	0.00	0.00	0.00	0.49	0.00
Bifidobacteriaceae (122)	0.00	0.00	0.00	-0.01	0.94
Bacteroides (166)	0.00	0.00	0.00	0.90	0.00
Lactobacillus fermentum (49)	0.00	0.00	0.00	-0.01	0.94
Bacteroides (445)	0.00	0.00	0.00	-0.01	0.94
Coprococcus (99)	0.00	0.00	0.00	-0.01	0.94
Bifidobacterium animalis (631)	0.00	0.00	0.00	1.00	0.00
Paraprevotella (508)	0.00	0.00	0.00	1.00	0.00

Supplementary Figure S1: nMDS plots. Non-metric multidimensional scaling (nMDS) plot based on Bray-Curtis (BC) dissimilarity between samples, visualizing the differences in overall gut microbiota community composition between sampling methods, per timepoint. Each data point represents the microbial community composition of one sample and is coloured by sampling method. The stress of the ordination was 0.280 (arbitrary). The effect sizes (R^2) and p-values calculated by permutational multivariate analysis of variance (PERMANOVA)-tests are shown in the plots. No significant differences in overall gut microbiota community composition between sampling methods were found.

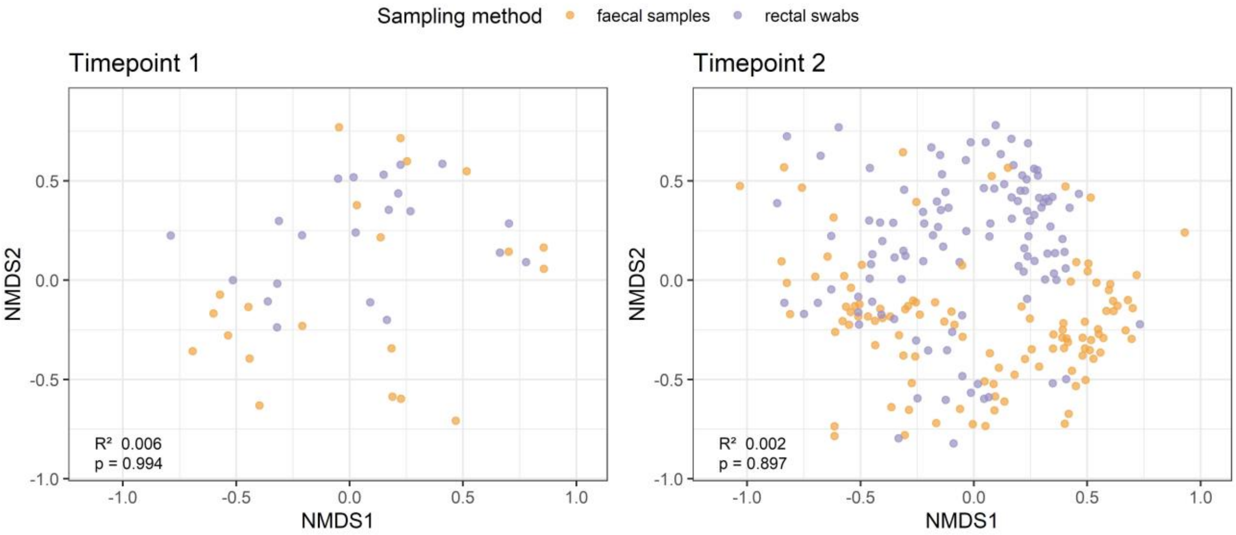

Supplement: Supplementary file 1 — Supplementary Information [file 41598_2019_52549_MOESM1_ESM.pdf]
